# The Parental Dilemma: How Evolution of Diverse Strategies for Infant Care Informs Social Behavior Circuits

**DOI:** 10.3389/fncir.2021.734474

**Published:** 2021-11-18

**Authors:** Anita E. Autry, Lauren A. O'Connell

**Affiliations:** ^1^Dominick P. Purpura Department of Neuroscience, Department of Psychiatry and Behavioral Sciences, Albert Einstein College of Medicine, Bronx, NY, United States; ^2^Department of Biology, Stanford University, Stanford, CA, United States

**Keywords:** parental care, neuroethology, neurogenetics, ethology, evolution

## Introduction

Social relationships are cornerstones of human well-being and functioning in society, which is painfully apparent during the current global pandemic that has forced social isolation. Indeed, one of the most urgent problems highlighted by this crisis is the lack of available childcare for working parents. We are far from understanding how behavior is governed by external and internal forces, despite decades of intense scientific and lay interest. Social behavior may be examined using a range of methods, and indeed we (the authors) have opposite, but converging, perspectives that complement one another and offer an opportunity to re-examine how the researchers approach the study of social behavior. One view is a “behavior-based” study of social behavior, which values an understanding of natural history, ethological variability, and ecology using field based studies that maximize comparative approaches through the lens of evolution. The other is a “brain-based” study of social behavior, which values an understanding of neuronal circuits, connectivity, or gene expression using laboratory based studies that minimize variability. We believe the lack of overlap or cross-talk between these approaches is a barrier for the growth of our field and here we argue that these approaches are complementary and require insights gained from one other.

Among a range of naturalistic behaviors, parenting is exciting to study because these social relationships can be complex, multi-generational, and long-lasting. Parental care is also an evolutionary antecedent to other complex social behaviors, such as monogamy and eusociality (Queller, 1994; Numan and Young, 2016). Thus, exploring parenting can lead to mechanistic insights that are broadly applicable to many aspects of social behavior. Here, we use parenting as an example of how “behavior-based” and “brain-based” approaches to studying behavior can yield insights both into generalizable principles of how organisms function and alternative neural mechanisms of behavior that give rise to a diversity of behavioral strategies.

## From Different Viewpoints: “Behavior-Based” vs. “Brain-Based”

Parental care has evolved independently across many taxa, highlighting the breadth of this behavior and the various ecological pressures that make this behavior more likely to increase offspring fitness. Parenting generally refers to behaviors that directly or indirectly benefit the young (Royle et al., 2012). For example, direct caregiving can consist of cleaning eggs, provisioning food to the young, carrying, retrieving, or grooming neonates, and huddling over newborns. Furthermore, indirect parenting behavior can include building and maintaining a nest, providing food for another parent, as well as protection of the young through defensive behaviors. While females are the sole providers of care in the majority of mammals, males play more prominent roles in other vertebrate taxa, where most birds display biparental care and amphibians and fishes show a greater flexibility in who cares for offspring. Interestingly, social monogamy co-occurs with male involvement in offspring care in many birds and mammals, highlighting how ecology and neural mechanisms are likely intertwined to facilitate paternal care. The repeated evolution of parental behavior with many different forms begs the question of whether there are ecological and neural commonalities across these independent origins or whether different neurobiological mechanisms drive similar behavioral forms.

Investigating parental care with an ethological and evolutionary lens often centers around asking why such a behavior exists (its adaptive significance and how it evolved) and how such a behavior is regulated by physiology (from an ontogenetic and mechanistic perspective) (Tinbergen, 1969). Variations in social and parental care strategies are tightly linked to ecological resources in their environment (Wilson and Landry, 1975), making field studies an important part of understanding behavioral evolution. For example, the evolution of biparental care in burying beetles (*Nicrophorus*) is thought to have evolved in part due to the limited availability of carcasses in which they raise their families (Scott, 1998). Another example comes from poison frogs (Family Dendrobatidae), which have diversified in parental care strategies based partly on the size of nursery utilized to rear their young (Brown et al., 2008; Furness and Capellini, 2019). In most dendrobatid species, males guard egg clutches and then transport hatched tadpoles to pools of water. Female involvement in offspring care evolved twice within this clade when species shifted to tiny pools of water to avoid tadpole predation (Summers et al., 2008), which necessitated mothers to provision tadpoles with food in these resource-poor nurseries. Understanding the ecological challenges and opportunities that species face in their natural habitat is key to understanding why certain parental care strategies have (or have not) independently evolved or diversified among closely related species or species occupying similar habitats.

Repeated evolution or rapid diversification of parental care strategies sets the stage for comparative work on how behavior is governed at more mechanistic levels. This kind of research embraces variation within and across species at deep and shallow phylogenetic levels. Recently, RNA sequencing has been used to examine how brain gene expression changes with the onset of parental behavior in burying beetles (Parker et al., 2015), earwigs (Wu et al., 2020), stickleback fish (Bukhari et al., 2019), and poison frogs (Fischer and O'Connell, 2020), all highlighting key metabolic or immune system pathways that shift in gene expression as individuals transition to parenthood. More sophisticated sequencing approaches like phosphoTRAP, which allows the isolation of mRNA from active neurons (Knight et al., 2012), is becoming increasingly used in unusual animals to narrow down thousands of differentially expressed genes to a few hundred that could be targeted in functional manipulations. Although phosphoTRAP has been used to identify transcripts enriched with paternal behavior in dendrobatid poison frogs (Fischer et al., 2019), it lacks the cellular resolution of single cell sequencing. However, single cell sequencing requires well-annotated genomes and thus its use will lag behind genome sequencing efforts. Thus, the embrace of breadth often comes at the cost of depth in probing neuronal circuits, as many technologies used to functionally manipulate neural circuits are species specific (e.g., most viral vectors optimized for expression in mice) or are difficult to implement in most freely moving animals (e.g., electrophysiological recordings of neural activity). This lack of cross-species neurogenetic technologies requires one to either build the tools from scratch or ask questions that can be answered by measuring gene expression or neural activity and conducting follow-up pharmacological manipulations.

Modern neuroscience tools have enabled us to build on this critical foundation of behavior-based knowledge to dissect the underlying neurobiology in the context of laboratory experiments. Typically in these studies, a population of neurons is first identified and then it is subsequently asked how these cells contribute to behavior based on neuromodulatory pathways and neuronal connectivity. The advantage of this approach is that the role of a specific population of neurons may be studied and dissected, but the downside is that other potentially critical or redundant components of the behavior circuit may be overlooked. However, the concept of a neural circuit implies the activity of multiple neural populations, from the sensory inputs to integration with interoceptive cues followed by motor outputs and recurrent feedback. Thus, a single neural population or even two nodes of a circuit may function as part of numerous behavior circuits. As a result, some neural circuit manipulations may lead to behavioral impacts that are somewhat contradictory or complex. Therefore, it is important to connect the physiological properties of a cell type or population as precisely as possible with the manipulations being performed. Specifically with regard to dissecting circuits for complex behaviors such as social interactions, it is key to uncover how neurons respond to discrete aspects of the behavior including locomotion, novelty, social cues such as odor or vocalizations, as well as the motor output components.

Social behavior poses a unique challenge for quantification and manipulation of neural circuits in the laboratory due to the freely moving nature of the interactions. This is particularly the case for parental behavior which relies on interactions between adults and offspring in a simulated naturalistic environment. The advent of less invasive tools for these applications is opening up the possibilities for studying parenting. For example, neuropixel probes allow high-density electrophysiological recordings from multiple brain sites over protracted time periods (van Daal et al., 2021). Lightweight, wireless devices are also becoming available for optogenetic and pharmacological manipulations as well as recording neural signals, enabling greater flexibility in studying social behaviors (Barbera et al., 2019; Zhang et al., 2019). Finally, the ever-decreasing size of implants required for recording, combined with increasing ability to multiplex, will allow for recording and/or manipulating from multiple brain regions and allow a more fully integrated understanding of how neural circuits coordinate behavior (Yamawaki et al., 2016; Meng et al., 2018; Jennings et al., 2019). However, one caveat is that the majority of these tools have been designed for use in mice and rats, which limits comparative studies (Stagkourakis et al., 2020). Another class of techniques propelling the field forward are single cell and single nucleus sequencing techniques which have revealed novel relationships among neural classes and are beginning to distinguish common and divergent genetic features among neurons active during specific behaviors (Moffitt et al., 2018; Lee et al., 2019). These approaches will lead not only to a more sophisticated understanding of neuronal cell-types involved in specific aspects of social behavior, but also enable more refined circuit manipulations using intersectional genetic targeting.

## Integrating Perspectives Moving Forward

Understanding parenting behavior from both ethological and neurogenetic viewpoints is transforming how we understand circuit dynamics and trade-offs in behavior. While the preponderance of previous research has focused on studying the neural control of parenting behavior, overwhelmingly in mothers, we have an opportunity to learn about neural circuits for infant care by studying behavior in non-parents, including male and female alloparental behavior or even infanticide (Lukas and Huchard, 2014; Rogers and Bales, 2019). For example, ethologists have long studied parental care trade-offs associated with the dilemma between choosing offspring investment or infanticide (Hausfater and Hrdy, 2017; Ringler et al., 2017). Infanticide often occurs to destroy the young of a competitor, such as in male lions (Packer and Pusey, 1983) or poison frogs (Ringler et al., 2017). When environmental resources are scarce, parents may also choose to invest in specific offspring to the harm of others, like when poison frogs feed their younger tadpoles to older ones (Rojas, 2014). Neurogenetics experiments have shed light on the reciprocal interactions of care and infanticidal circuits, where circuit nodes highly active during parental care are often silent in infanticidal animals and vice versa, suggesting that the behavior circuits may be intertwined in order to tightly control the expression of behavior (Wu et al., 2014; Odaka et al., 2015). In mice, beautiful neurogenetics work has highlighted galanin as promoting parental care and inhibiting aggression (Wu et al., 2014; Kohl et al., 2018), while urocortin-3 seems to promote infanticide (Autry et al., 2021). In turn, these neurogenetics experiments have inspired more ethological work that has found that galanin contributes to parental care across a wide range of taxa (Fischer et al., 2019; Butler et al., 2020), but not all (Tripp et al., 2020). Thus, it appears as though highly conserved galanin circuits across animals may be evolutionarily tuned to promote the gains and losses of parental care in various taxa.

Moving forward, we call for more integration of ethological and neurogenetics approaches, whose reciprocal feedback enriches both the breadth and depth at which we understand the mechanisms of behavior and how these evolve and diversify. Neurogenetics allows extreme precision in understanding neural circuits while comparative work is necessary for understanding what neural circuit principles are generalizable across taxa vs. specific to a subset of species. Ultimately, it is behavior and physiology that are the substrates of natural selection and therefore understanding behavior in the context of the natural environment will be key to deciphering the purpose of any neural circuit. We believe that integration of both ethological and neurogenetic perspectives will open new experimental avenues, better define questions, and refine experimental approaches moving forward.

## Author Contributions

All authors listed have made a substantial, direct and intellectual contribution to the work, and approved it for publication.

## Conflict of Interest

The authors declare that the research was conducted in the absence of any commercial or financial relationships that could be construed as a potential conflict of interest.

## Publisher's Note

All claims expressed in this article are solely those of the authors and do not necessarily represent those of their affiliated organizations, or those of the publisher, the editors and the reviewers. Any product that may be evaluated in this article, or claim that may be made by its manufacturer, is not guaranteed or endorsed by the publisher.
